# Safely Discharging Infants with Bronchiolitis from an Emergency Department: A Five Step Guide for Pediatricians

**DOI:** 10.1371/journal.pone.0163217

**Published:** 2016-09-30

**Authors:** Fabiola Stollar, Alain Gervaix, Constance Barazzone Argiroffo

**Affiliations:** 1 General Pediatric Division, Children’s Hospital, University Hospitals of Geneva, Geneva, Switzerland; 2 Pediatric Emergency Division, Children’s Hospital, University Hospitals of Geneva, Geneva, Switzerland; 3 Pediatric Pulmonology Unit, Children’s Hospital, University Hospitals of Geneva, Geneva, Switzerland; University Children's Hospital Bern, SWITZERLAND

## Abstract

Recent publications have established the pulse oxygen saturation (SpO_2_) threshold of 90% for the hospitalization and discharge of infant patients with bronchiolitis. However, there is no clear recommendation regarding the Emergency Department (ED) observation period necessary before allowing safe home discharge for patients with SpO_2_ above 90%-92%. Our primary aims were to evaluate the risk factors associated with delayed desaturation in infants with SpO_2_ ≥ 92% on arrival at the ED as well as the ED observation period necessary before allowing safe home discharge. A secondary aim was to identify the risk factors for ED readmission. Of 581 episodes of bronchiolitis in patients < 1 year old admitted to the ED, only 47 (8%) had SpO_2_ < 92% on arrival there, although 106 (18%) exhibited a delayed desaturation (to < 92%) during ED observation. Female sex, age < 3 months old, ED readmission, more severe initial clinical presentation, and higher pCO_2_ level (> 6KPa) were risk factors for delayed desaturation with OR varying from 1.7 to 7.5. In patients < 3 months old, mean desaturation occured later than in older patients [6.0 hours (IQR 3.0–14.0) vs. 3.0 hours (IQR 2.0–6.0), *P* = 0.0018]. In 95% of patients with a delayed desaturation this decrease occurred within 25 hours for patients < 3 months old and within 11 hours for patients ≥ 3 months old. In patients < 3 months old with respiratory rates above the normal range for their age the desaturation occurred earlier than in patients < 3 months with normal respiratory rates [4.4 hours (IQR 3.0–11.7) vs. 14.6 hours (IQR 7.6–22.2), P = 0.037]. Based on the present study’s results, we propose a five step guide for pediatricians on discharging children with bronchiolitis from the ED. By using the threshold of an 11 hour ED observation period for patients ≥ 3 months old and a 25 hour period for patients < 3 months old we are able to detect 95% of the patients with bronchiolitis who are at risk of delayed desaturation.

## Introduction

Although bronchiolitis is the leading cause of hospitalization for infants with an estimated 3.4 million admissions globally every year [1], most children arriving at an emergency department (ED) with bronchiolitis are discharged home after their medical consultation [2,3]. However, even in children with an apparently mild disease, the clinical course is often unpredictable, with the risk of subsequent worsening making it difficult for physicians to determine the appropriate observation period and setting [4]. According to the latest American Academy of Pediatrics guidelines and to other recent publications, the pulse oxygen saturation (SpO_2_) threshold of 90% has been established as one of the criteria for hospitalization [2] and for safe discharge after hospitalization [5,6]. Swiss guidelines recommend hospitalization and oxygen supplementation for SpO_2_ < 92% [7]. However, there is no clear recommendation regarding the ED observation period necessary before allowing safe discharge home for patients with SpO_2_ above 90%-92%.

Indeed, the risk of hospital-acquired infection and the costs associated with unneeded treatments and hospitalization could be avoided if low-risk children could be safely discharged home. There is a lack of studies evaluating predictors of unnecessary hospitalization and safe discharge for this common disease. Mansbach *et al*. created a low risk model including cut-off points for age, respiratory rate (RR) and oxygen saturation with which to identify children who might experience an unanticipated clinical deterioration and a more severe outcome [8]. However, another retrospective study found that oxygen saturation and clinical assessment failed to differentiate between patients with bronchiolitis who had consulted a second time and required hospital admission, and patients who had not [9]. Previous studies have not provided information about the ideal observation period necessary before considering an ED discharge to be without risk.

Existing data are still controversial, and there is no clear recommendation on how to manage children experiencing a more severe episode, but who do not fulfill all the hospitalization criteria. We aimed to clarify these uncertainties and help pediatric emergency physicians in their clinical decision making.

Our primary aims were to evaluate the risk factors for delayed desaturation in patients with initial SpO_2_ levels within normal limits on arrival at the ED and to define the ED observation period required before allowing safe discharge home. A secondary aim was to identify the risk factors for ED readmission.

## Methods

### Study Design

The present study was a retrospective medical chart review conducted at the University Children’s Hospital of Geneva–a tertiary care level hospital in Switzerland.

During two respiratory syncytial virus (RSV) seasons (2010–2012), children were eligible for inclusion in the study if they were aged less than 1 year old and their primary or secondary discharge diagnosis was bronchiolitis (*International Classification of Diseases*, *version 2010*, codes: J21, acute bronchiolitis; J21.0, acute bronchiolitis due to RSV; and J21.9, acute bronchiolitis, unspecified) identified from computerized hospital charts. The diagnosis of bronchiolitis was based on the clinical presentation associating respiratory distress, crackles, and wheezing [2]. Patients with chronic diseases such as congenital heart disease, genetic disorders, bronchopulmonary dysplasia, congenital or acquired immunodeficiencies, and neuromuscular disorders were excluded. Premature infants without chronic diseases were not excluded. We analyzed a subgroup of patients aged < 3 months old because such infants are at a greater risk of developing a more severe course of disease [2]. This was a convenience sample.

This study was approved by the institution’s ethical committee on clinical research in children—Comité d’Ethique des Hôpitaux Universitaires de Genève, Suisse (protocol 12–026). Patient records/information was anonymized and de-identified prior to analysis.

### Data Collection

Hospital charts, together with information gathered from a hospital patient information database, were used to extract data for predefined data collection sheets, including: demographic and epidemiological characteristics (age, sex, gestational age, and date of admission); medical history (history of wheezing); underlying medical conditions (prematurity: < 37 weeks of gestation, and chronic diseases); radiological (chest X-ray) and laboratory tests (capillary blood gas analysis); disease-severity parameters (mortality, hospitalization, ED observation period, hospital length of stay (LOS), intensive care unit (ICU) admission, requirement and duration of enteral feeding, supplemental oxygen, and mechanical ventilation).

Capillary blood gas analysis, measuring the partial pressure of carbon dioxide (pCO_2_), was recorded only if performed during the first 6 hours after ED admission. For the present analysis, pCO_2_ > 6 kPa was considered an abnormal level.

Patient’s ED charts provided the following clinical data: RR; presence of wheezing; degree of retractions [divided for this analysis into none/mild: none or retraction in only one site (e.g., subcostal retraction or nasal flaring) vs. moderate/severe: retractions in more than on site (e.g., supraclavicular and sternal retractions)]; presence or absence of feeding difficulties; cyanosis and apnea; clinical management; and hospital setting.

SpO_2_ levels were analyzed and three groups were defined: 1) patients without desaturation < 92% during their hospital stay; 2) patients with SpO_2_ < 92% on ED arrival; 3) patients with SpO_2_ ≥92% on ED arrival but that dropped below 92% at any time during their stay. The latter group was defined as having a delayed desaturation.

For this analysis and to avoid any overlap between a new episode of bronchiolitis and a second ED consultation for the same episode, we defined an ED readmission as a child consulting urgently at the ED for worsened symptoms of bronchiolitis within 7 days of the first ED presentation.

The hospital LOS and ED observation period were obtained from administrative data (recorded automatically by the hospital computer systems): the time at hospital or ED discharge minus the time in ED triage were used to calculate the LOS to the nearest hour. For each patient, the beginning and resolution times of any supportive therapies (oxygen supplementation or nasogastric feeding tube) were identified and their durations calculated.

### Standard of Care

In our hospital we used the following protocol for hospitalization criteria [7]: oral intake below 50% of the daily required amount, SpO_2_ < 92% in room air, progressive respiratory failure, apnea, bradycardia, and poor social conditions. Discharge criteria were: oral intake above 50% of the daily required amount, absence of vomiting, and SpO_2_ ≥ 92% in room air during an undisrupted sleep.

Infants admitted during the study period were provided with supportive care. They were nourished via nasogastric tube when intake was < 50% of normal or when excessive work of breathing was noted. Supplemental oxygen was administered through nasal cannula if SpO_2_ was < 92% in room air. Other treatments, such as inhaled β-receptor agonist or oral steroids were discouraged as shown to be ineffective [2,10]. Antibiotics were only administered when a concomitant bacterial infection (e.g., pneumonia, acute otitis media) was suspected. Oxygen therapy was stopped once SpO_2_ levels in room air were ≥ 92% during an undisrupted sleep. SpO_2_ was continuously monitored using a Philips model M3 oximeter.

### Statistical Analysis

All analyses were performed using STATA version 12.0 software (StataCorp, College Station, TX). Results for categorical variables are presented as proportions with 95% confidence intervals (CI) for an odds ratio (OR). Results for continuous variables are presented as a mean with standard deviation (SD) or as a median with interquartile range (IQR).

The risk factors associated with delayed desaturation were evaluated. Infants with delayed desaturation and a subsequent need of oxygen therapy were compared to infants discharged home from the ED. We analyzed the interval between the ED arrival time and the time to a drop in the SpO_2_ level. We performed univariate analyses using Student’s t-test, the Mann-Whitney test, the chi-square test or Fisher’s exact test, as appropriate. The factors associated with an increased risk of delayed desaturation were included in a multivariate model. We also evaluated the risk factors associated with ED readmission.

Linear regression (Pearson’s correlation coefficient) was used to analyze correlations between the tests. We analyzed the correlation between the duration of symptoms before ED arrival and the time taken for SpO_2_ to decrease. All *P* values were two-tailed, with *P* < 0.05 considered as statistically significant.

## Results

### Study Group

Of the 546 infants presenting with bronchiolitis to the ED during the study period, 478 (161 female: 317 male) under one year old and without chronic diseases were included, corresponding to 581 episodes. This included 21 (4%) premature infants without chronic diseases. A mean of 1.3 ± 0.7 episodes per patient was noted. The mean age was 5.85 ± 3.2 months old (range, 0.3–12.0), however hospitalized patients were younger than non-hospitalized ones (4.4 vs. 6.5 months old, P < 0.001). Of the 184 (31.6%) hospitalizations recorded, 17 (9%) went to the ICU and 167 (91%) went to wards. The mean LOS was 4.8 ± 3.4 days. No deaths were documented. Four patients presented apnea (only 1 was < 3 months old). Table 1 compares the clinical characteristics of patients <and ≥ 3 months old.

**Table 1:** Characteristics of study patients and outcome according to age.

	< 3 months (n = 137)	≥ 3 months (n = 444)	OR (95% CI)	*P* value
Female sex, %	42	30	1.7(1.1–2.5)	0.01
Premature,%	2	7	0.3(0.08–0.9)	0.04
Duration of symptoms at presentation, median(IQR), days	2(1–3)	2(1–3)	_	0.5
ED readmissions, %	5	7	0.7 (0.3–1.6)	0.38
Respiratory rate above normal range for age, %^¥^	71	63	1.4(0.9–2.1)	0.101
Moderate/severe retractions, %	58	52	1.(0.9–1.9)	0.19
SpO_2_ at arrival, mean±SD, %	96±3.8	96±4.5	_	0.96
Observation period in ED, median(IQR), hours	4.1 (2.8–5.8)	3.6(2.4–5.5)	_	0.017
Required oxygen therapy, %	49	19	4(2.7–6.1)	<0.001
Required NGT, %	40	7	8.5(5.2–14)	<0.001
Hospitalized patients, %	65	21	6.7(4.4–10.1)	<0.001
Length of hospital stay, median(IQR), days	4.8(3.1–6.3)	3.2(2.0–4.7)	_	<0.001
Duration of oxygen therapy, median(IQR), days*	3.0(1.6–4.8)	1.7(0.6–2.9)	_	<0.001
Duration of NGT, median(IQR), days ^§^	2.9(1.9–6.1)	2.8(1.6–3.9)	_	0.45
Capillary blood gas analysis episodes n(%)	41(30)	36(8)	4.8(2.9–7.9)	<0.001
pCO_2_ > 6 KPa	56	6	21.7(4.6–102.7)	<0.001

NGT, nasogastric tube; IQR, interquartile range; CI, confidence interval; OR, odds ratio; ED, emergency department; SpO2, pulse oxygen saturation.

¥ normal values for age groups as follows: 0–1.9 months old, 45 breaths/minute; 2–5.9 months old, 43 breaths/minute; 6–11.9 months old, 40 breaths/minute

* n = 149 (67episodes < 3 months)

§ n = 84 (54 episodes < 3 months)

### Risk Factors Associated with Delayed Desaturation

**Fig 1:**
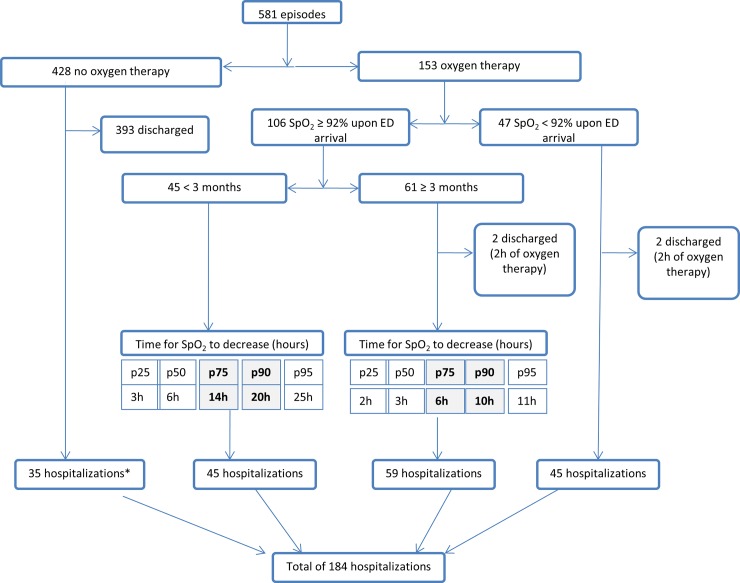
Patients’ outcomes from ED arrival until admission to the hospital or discharge home. *35 patients did not need oxygen therapy but were hospitalized for enteral feeding, social reasons, or observation due to important respiratory distress.

Of 581 episodes analyzed, 428 (74%) had SpO_2_ ≥ 92% at ED arrival and never required supplemental O_2_; 153 (26%) did require supplemental O_2_ because of SpO_2_ < 92%. Among these 153 episodes, only 47 (30%) had SpO_2_ < 92% at ED arrival and 106 (70%) had a delayed desaturation after a few hours of observation “Fig 1”. Considering all episodes, female sex, age < 3 months old, ED readmission and a more severe initial clinical presentation (higher pCO_2_, RR above the normal range for the age, and moderate/severe retractions) were associated with an increased risk for a delayed desaturation, with odds ratios varing from 1.7 to 7.5. In infants < 3 months old, only female sex, moderate/severe retractions, and higher pCO_2_ levels remained statistically significant. Because capillary blood gas analysis was only performed on a small number of children (n = 58), the resulting estimate was unreliable and therefore not included in the multivariate analysis "Table 2".

**Table 2:** Risk factors for delayed desaturation (SpO_2_ < 92%).

Patients ≥ and < 3 months old (n = 534)							< 3 months (n = 116)			
Predictor	No decrease in SpO2 (n = 428)	Delayed decrease in SpO_2_ (n = 106)	OR (95% CI)	Univariate analysis	Adjusted OR (95% CI)	Multivariate analysis	OR (95% CI)	Univariate analysis	Adjusted OR (95% CI)	Multivariate analysis
				*P*		*P*		*P*		*P*
Age < 3 months old	16%	43%	3.8(2.4–6.1)	< 0.001	4.2(2.6–6.9)	< 0.001	_	_	_	_
Female sex	30%	42%	1.7(1.1–2.6)	0.015	1.7(1.0–2.7)	0.036	2.4 (1.1–5.1)	0.027	2.4(1.1–5.3)	0.036
pCO_2_ > 6 kPa ∞	11%	48%	7.5 (1.8–30.2)	0.005	n.a.	n.a.	5.3 (1.1–26.6)	0.041	n.a.	n.a.
Moderate/severe retractions	45%	69%	2.75(1.7–4.3)	< 0.001	2.6(1.6–4.3)	< 0.001	2.9 (1.3–6.4)	0.007	2.9(1.3–6.1)	0.009
RR > normal for age*	58%	78%	2.4(1.5–4.0)	< 0.001	1.9(1.1–3.2)	0.017	1.9(0.9–4.6)	0.24	_	_
ED Readmission	4%	12%	3 (1.4–6.2)	0.0039	3.6(1.6–8.1)	0.002	3.1(0.3–35)	0.59	_	_

CI, confidence interval; OR, odds ratio; RR, respiratory rate; SpO2, pulse oxygen saturation; ED, emergency department.

*Normal values for age groups as follows: 0–1.9 months old, 45 breaths/minute; 2–5.9 months old, 43 breaths/minute; 6–11.9 months old, 40 breaths/minute.

∞ Capillary blood gas analysis was performed in 58 episodes (32 episodes in children < 3 months old). Duration of symptoms more than 2 days, prematurity, wheezing, cyanosis, atelectasis, β2 agonists in the hospital, repeated episodes, and personal allergy were not significant for all patients or for patients < 3 months old.

### ED Observation Period to Allow Safe Discharge

The mean ED observation period for infants with bronchiolitis was 5 ± 4 hours with a median of 3.7 hours (IQR: 2.5–5.6). “Fig 1” shows an algorithm of the patients’ course from ED arrival until admission to hospital or discharge home.

In the 106 episodes where a delayed desaturation was observed, the initial clinical presentation was similar for all patients, whether < and ≥ 3 months old, except for the SpO_2_ at ED arrival which was significantly higher in patients < 3 months old (97 ± 2 *vs*.95 ± 2; P < 0.001) "Table 3". However, the time to desaturation did vary with age; patients < 3 months old desaturated later than patients ≥ 3 months old [6.0 hours (IQR 3.0–14.0) vs. 3.0 hours (IQR 2.0–6.0), *P* = 0.0018]. In 95% of the patients, the delayed desaturation occurred within 25 hours for patients < 3 months old and within 11 hours for patients ≥ 3 months “Fig 1”.

**Table 3:** ED presentation in patients with delayed desaturation (SpO_2_ < 92%) according to age.

	< 3 months (n = 45)	≥ 3 months (n = 61)	OR (95% CI)	*P* value
Duration of symptoms at presentation, median(IQR), days	2(1–4)	2 (1–3)	_	0.51
ED readmission, %	4	18	0.2(0.04–0.9)	0.048
Respiratory rate above normal range for age, %	78	79	0.9(0.3–2.1)	0.75
Retractions moderate/severe, %	69	70	0.9(0.4–2.0)	0.73
SpO_2_ at arrival, mean±SD, %	97 ± 2	95 ± 2	_	< 0.001
Time to desaturation, median (IQR) hours	5.9(3.0–13.8)	3.3(1.9–5.9)	_	0.0018

IQR, interquartile range; CI, confidence interval; OR, odds ratio; SpO_2_, pulse oxygen saturation; ED, emergency department

The only risk factor for a faster desaturation was a RR above the normal range for the age. Patients < 3 months old with a RR above the normal range for the age were more likely to experience desaturation earlier than patients < 3 months with a normal RR (4.4 hours (IQR 3.0–11.7) vs. 14.6 hours (IQR 7.6–22.2), *P* = 0.037). Female sex, moderate/severe retractions and ED readmission were not risk factors for a faster desaturation.

**Fig 2:**
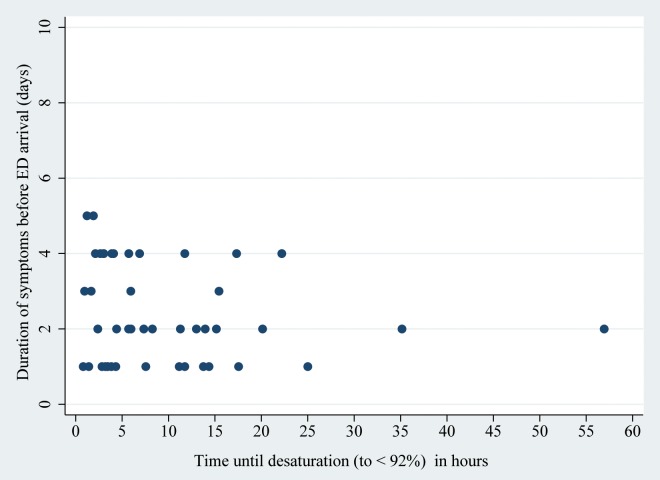
Correlation between duration of symptoms before Emergency Department (ED) arrival and time until desaturation (to < 92%) in patients < 3 months (n = 45). R^2^ = 0.02, *P* = 0.32.

**Fig 3:**
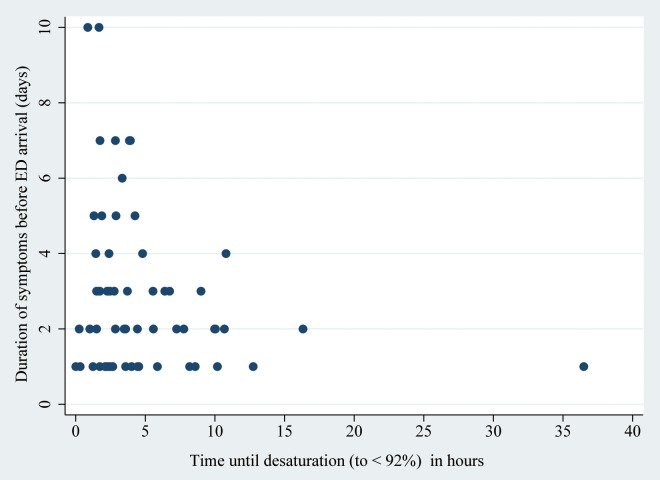
Correlation between duration of symptoms before Emergency Department (ED) arrival and time until desaturation (to < 92%) in patients ≥ 3 months (n = 61). R^2^ = 0.05, *P* = 0.08.

We therefore evaluated whether the time to desaturation was influenced by the duration of the symptoms before ED arrival and, interestingly this was not the case ("Figs 2 and 3").

### Risk Factors for ED Readmission after Initial ED Discharge

In this cohort of patients, 36/478 (7.5%) of those discharged home from the ED required second ED consultation within 7 days. Infants with a RR above the normal range for their age were more likely to be readmitted (OR 2.5; 95% CI: 1.1–5.4). Prematurity, age, sex, a previous episode, and the severity of retractions were not risk factors for ED readmission.

Four patients were not hospitalized but needed oxygen therapy in the ED for a mean of 2 ± 1 hours because of SpO_2_ < 92% at ED arrival or during their ED stay; they were discharged after a mean 8.4 ± 3.4 hours of observation. Of these patients, only one female aged ≥ 3 months old needed ED readmission.

## Discussion

A decrease in oxygen saturation is one of the most important factors for hospitalization and the most common barrier to discharge home for infants with bronchiolitis [8,11]. In the present study, it is interesting to note that most of the infants (70%) hospitalized for oxygen supplementation arrived at the ED with a normal SpO_2_ (≥ 92%), and showed a delayed desaturation during their ED stay. This important finding highlights the need to identify the risks factors for delayed desaturation and standardize management procedures in EDs.

Our study provides new information on children’s clinical profiles associated with risk of delayed desaturation. We found that female sex, moderate/severe retractions and a RR above the normal range for the age on arrival at the ED, age < 3 months old, ED readmission, and increased pCO_2_ were significantly associated with this delayed desaturation.

Previous studies described that younger age and a more severe initial clinical presentation were risk factors for hospitalization and a more severe clinical course [3,12]. However, other studies showed that the usefulness of clinical severity scores for predicting an oxygen requirement was limited and that oxygen saturation and age did not predict which children would be appropriate candidates for admission to short-stay observation units [13,14].

In contrast to our findings, some authors found that male sex was a risk factor for a more severe disease course [3,4]. However, other studies did not find sex to be associated with either an increased risk of ICU admission or safe discharge home from the ED [8,15]. The reasons for these seemingly discrepant results are unclear.

Mulholland *et al*. concluded that SpO_2_ and arterial pC0_2_ levels at ED admission were the more accurate measures for predicting the need for a high fraction of inspired oxygen [16]. The latest studies have found that capnometry readings upon ED arrival did not predict hospital admission or discharge from ED and that end-tidal CO_2_ was not correlated with disease severity measures [17,18]. The present study suggests that capillary blood gas analysis may help clinicians to detect patients at risk of a delayed desaturation and accordingly, patients with a pCO_2_ > 6 kPa were more likely to experience desaturation. We did not perform arterial blood gas analysis, and indeed this procedure is not usually recommended in the initial evaluation of bronchiolitis. Moreover, in a meta-analysis comparing arterial versus capillary blood gas analysis, the authors concluded that sampling blood from the fingertip or earlobe reflects arterial pCO_2_ and pH accurately over a wide range of values [19]. We are aware that only a few infants underwent blood gas analysis and the present results require validation in a larger group of patients.

Similar to Mansbach *et al*. [20] who found a median ED observation period of 3.3 hours (IQR: 2.3–4.8 hours) in our study the median ED observation period was 3.7 hours (IQR: 2.5–5.6 hours). Some infants had a much later delayed decrease of SpO_2_ and might have been maintained in the ED for other reasons. A clinical evaluation is, therefore, mandatory for judging a potential discharge, and assessment of the SpO_2_ level is a useful adjunct [21].

One recent study showed that children with mild bronchiolitis presented short recurrent desaturations or sustained desaturations after discharge home. However, the authors did not assess the time for a desaturation in patients with a more severe disease [22]. To the best of our knowledge, the time to a delayed desaturation and the ideal length for an ED observation period have never before been specifically assessed for patients with a severe bronchiolitis and an initial normal SpO_2_ level. The novelty of this study is that it provides physicians with an initial decision-making rule for this patient group, with which to decide on safe discharge home or hospital admission.

Our findings suggested that most of the children arriving at the ED with mild to moderate bronchiolitis maintained normal oxygen saturation. In patients who subsequently required oxygen supplementation, 95% of patients < 3 and ≥ 3 months old experienced desaturation within 25 hours and 11 hours of ED arrival, respectively.

However, the present study’s most surprising result was that patients < 3 months old took twice as long to experience desaturation (SpO_2_ < 92%) compared to patients ≥ 3 months old. Interestingly, we also found that patients < 3 months old with a RR above the normal range for that age showed decreased in SpO_2_ levels earlier than patients < 3 months old with a normal RR (4.4 vs. 14.6 hours), however the time to desaturation was not influenced by the severity of retractions. We could speculate as to why desaturation occurs earlier when a child < 3 months old arrives with a RR above the normal range for its age. This may be due to the fact that in young infancy the RR is closely related to the severity of the disease. It is known that tachypnea in young children is one of the earliest compensatory mechanisms for inadequate ventilation [23]. Indeed, a newborn’s ribs come off the spine more horizontally than on older children and, therefore, cannot be lifted to increase the tidal volume. This could explain why newborns are dependent on an increased RR rather than on tidal volume to compensate for respiratory impairment [24].

Furthermore, clinical signs of respiratory distress may not capture the hypoxemia/hypercapnia balance equally. Fernandes *et al*. found a weak negative correlation between baseline Respiratory Distress Assessment Instrument scores and SpO_2_ levels (Spearman’s r = 20.24; P < 0.001; n = 1761) [25]. The authors argued that their results were different from others because their predefined cut-offs may have been too strict. Furthermore, they did not analyze a subgroup of patients < 3 months old, as we did.

The present study also analyzed the possibility that the longer the symptoms had existed prior to ED arrival, the sooner the desaturation would happen. However, the findings could not confirm this hypothesis. We also hypothesize that the inherent severity of a case of bronchiolitis might change the time required for the SpO_2_ level to decrease and that clinical outcome depends more on the adaptive capacities.

The present study’s percentage of ED patients readmitted within 7 days was 7.5%, and the only risk factor for ED readmission was a RR above the normal range for the infant’s age. In a prospective cohort study, Norwood *et al*. described a higher rate of readmission of 17%. They found that the three independent factors associated with readmission were age < 2 months old, male sex, and a previous history of hospital admission. However, they defined readmission as an urgent visit to an ED⁄clinic for worsened bronchiolitis within 2 weeks of discharge. Nonetheless, their study described a median time for readmission of 2 days, and 65% of children relapsed within 2 days of their initial ED visit [4].

We propose a five step guide for pediatricians wishing to discharge children with bronchiolitis from an ED. Step 1: detect patients with risk factors for a delayed desaturation (age < 3 months old, ED readmission, and a more severe initial clinical presentation). Step 2: in patients at risk, we recommend an ED observation period of 11 hours for patients ≥ 3 months old and 25 hours for patients < 3 months old. Step 3: an infant can be discharged home if no desaturation happens within this period and the child is feeding well, however, the pediatrician should be aware that an elevated RR is a major risk factor for ED readmission. Step 4: provide anticipatory guidance instructions to the caregivers of those children at high risk; they should be advised to return for a reevaluation in cases of worsening respiratory distress or if the child is feeding less than 50% of the daily required amount. Step 5: the caregiver should bring the child to the ED for a reevaluation 12 hours after ED discharge, especially in younger patients (< 3 months old) and in ED readmissions. Steps 4 and 5 are relatively subjective and are good practice suggestions; steps 1–3 are based on our results.

This five step guide does not suggest measuring oxygen saturation at home because its interpretation is not always easy, particularly in cases of bronchiolitis where brief, frequent desaturations to < 90% while sleeping are frequent [26–28]. Decisions based on pulse oximetry increase rates of hospitalization for bronchiolitis [21] and it is not an effective tool for predicting subsequent readmission for care [22]. Furthermore, this was not within the scope of the present study.

Following the steps described above, 95% of patients with bronchiolitis and at risk of a delayed desaturation will be detected. With thresholds of 10 hours for patients ≥ 3 months old and 20 hours for patients < 3 months old, 90% will be detected. Depending on the availability of observation beds in an ED (season, hospital crowding), pediatricians may have to be more or less permissive and choose the observation period most convenient for their own institution (“Fig 1”: p75, p90 or p95).

Future prospective, multicenter studies on the ideal ED observation period in terms of feasibility should compare different lengths of observation based on the present study’s results and then evaluate patient safety versus the costs and availability of shorter and longer evaluations.

This study’s limitations include its retrospective component and cohort selectivity limited by the International Classification of Diseases, thus some cases may have been missed. Secondly, we also included patients with a previous history of wheezing, and although some investigators emphasize the importance of looking at bronchiolitis only in those patients without a prior wheezing illness, this is not a consensus and heterogeneity in the population exists [2,29]. Thirdly, our study population consisted of children consulting at the ED of a tertiary hospital, with the consequent probability of greater disease severity. It is thus possible that our sample does not represent all infant bronchiolitis patients and its results are not necessarily generalizable to other clinical settings, such as primary care services. However, our study did also include many patients with a mild form of the disease, because the majority of them were discharged home. Lastly, the RR, SpO_2_, and degree of retractions used for analysis were the levels collected initially at the ED; throughout the ED observation period patients probably experienced changes in all these measures. Repeated observations over a longer period of time, rather than a single examination, would provide a more valid overall assessment. Indeed, the present study does not include information about the etiology of the infectious cause or vaccination status. However, in a previous study, we showed that virological testing does not help in clinical management decisions, and at an individual level, as a performance test, it seems insufficient to precisely predict outcomes [30].

Bronchiolitis, though a common cause of admission to ED observation units, is associated with a high rate of short-stay failure and continued inpatient care [31,32]. This suggests that it is important to find some means to facilitate early discharges and safely reduce the burdens of long ED observation periods.

## Conclusions

In conclusion, we propose a five step guide for pediatricians wishing to discharge infants with bronchiolitis from an ED. This may help clinicians in the management of children with bronchiolitis and reduce practice variability and the subsequent costs associated with hospitalizations for this common condition. These interesting results await further validation and analysis in prospective studies, especially cost effectiveness studies.
