# Correction: Safely Discharging Infants with Bronchiolitis from an Emergency Department: A Five Step Guide for Pediatricians

**DOI:** 10.1371/journal.pone.0166256

**Published:** 2016-11-02

**Authors:** Fabiola Stollar, Alain Gervaix, Constance Barazzone-Argiroffo

The third author's name was not hyphenated and the citation was incorrect. The correct name is: Constance Barazzone-Argiroffo.

The correct citation is: Stollar F, Gervaix A, Barrazone-Argiroffo C (2016) Safely Discharging Infants with Bronchiolitis from an Emergency Department: A Five Step Guide for Pediatricians. PLoS ONE 11(9): e0163217. doi:10.1371/journal.pone.0163217
